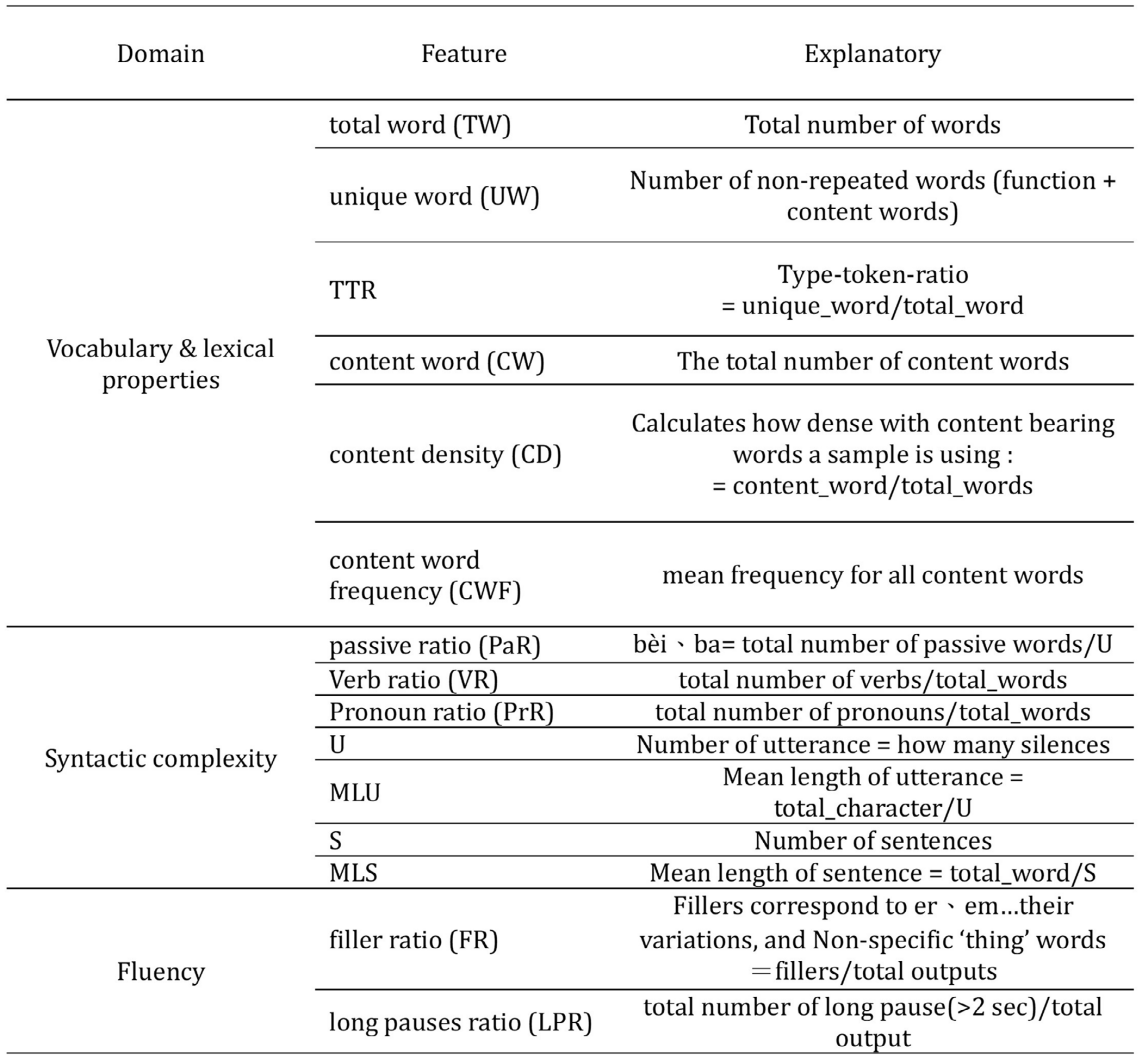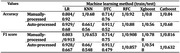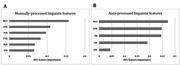# Contrasting Manual and Automatic Approaches for Extracting Linguistic Features in Predicting Alzheimer’s Disease through Chinese Speech

**DOI:** 10.1002/alz.088635

**Published:** 2025-01-03

**Authors:** Yi‐Hsuan Wang, Chia‐ Fang Cheng, Chia‐Ju Chou, Yi‐Chien Liu, Chia‐Ying Lee, Ya‐Ning Chang

**Affiliations:** ^1^ College of Medicine, National Cheng Kung University, Tainan Taiwan; ^2^ Miin Wu School of Computing, National Cheng Kung University, Tainan Taiwan; ^3^ Cardinal Tien Hospital, New Taipei City Taiwan; ^4^ Medical school of Fu‐Jen University, Taipei Taiwan; ^5^ Tohoku University, Sendai Japan; ^6^ Institute of Linguistics, Academia Sinica, Taipei Taiwan

## Abstract

**Background:**

Continuous speech analysis is considered as an efficient and convenient approach for early detection of Alzheimer’s Disease (AD). However, the traditional approach generally requires human transcribers to transcribe audio data accurately. This study applied automatic speech recognition (ASR) in conjunction with natural language processing (NLP) techniques to automatically extract linguistic features from Chinese speech data. Machine learning (ML) models were then used to predict early AD. The obtained results were compared with those derived from manual processes.

**Method:**

Continuous speech samples were obtained from 81 native Chinese speakers in Taiwan performing a picture description task. The participants included 34 normal controls (61‐89 yrs) and 47 patients diagnosed with amnestic mild cognitive impairment (aMCI) and early AD (59‐89 yrs). Fifteen linguistic features (Table 1) were measured via both manual and automatic processes. In the automatic extraction process, an ASR model transcribed the speech automatically, followed by the application of NLP techniques for automatic extraction of linguistic features. To classify AD, six ML models were trained with the two sets of features, and their performance was compared. The training‐to‐testing data ratio was 7:3, and cross‐validation was employed to mitigate overfitting.

**Result:**

The Random Forest classifier achieved the best prediction accuracy and F1 score in both the datasets processed manually (acc = 0.92, F1 score = 0.908) and automatically (acc = 0.88, F1 score = 0.857) (see Table 2). Figure 1 illustrates the top five linguistic features for both the manually‐processed (Figure 1A) and auto‐processed (Figure 1B) datasets. The results showed an overlap in three out of five key features were overlapped including mean length of utterance (MLU), long pause ratio and pronoun ratio.

**Conclusion:**

Our automatic approach for extracting linguistic features achieved comparable accuracy (88%) with those extracted through human judgment in Random Forest classifiers (92%). Importantly, MLU emerged as the predominant feature in both manually‐ and automatically‐processed datasets. This outcome suggests a noteworthy decline in syntactic complexity during the early stages of AD in Chinese‐speaking patients.